# *APOL1-G0* protects podocytes in a mouse model of HIV-associated nephropathy

**DOI:** 10.1371/journal.pone.0224408

**Published:** 2019-10-29

**Authors:** Leslie A. Bruggeman, Zhenzhen Wu, Liping Luo, Sethu Madhavan, Paul E. Drawz, David B. Thomas, Laura Barisoni, John F. O'Toole, John R. Sedor

**Affiliations:** 1 Departments of Inflammation & Immunity and Nephrology, Cleveland Clinic Lerner College of Medicine, Case Western Reserve University, Cleveland, Ohio, United States of America; 2 Department of Physiology and Biophysics, Case Western Reserve University School of Medicine, Case Western Reserve University, Cleveland, Ohio, United States of America; 3 Department of Medicine, Ohio State University, Columbus, Ohio, United States of America; 4 Department of Medicine, University of Minnesota, Minneapolis, Minnesota, United States of America; 5 Departments of Pathology, University of Miami, Miami, Florida, United States of America; 6 Departments of Pathology and Medicine, Duke University, Durham, North Carolina, United States of America; University of Houston, UNITED STATES

## Abstract

African polymorphisms in the gene for Apolipoprotein L1 (*APOL1*) confer a survival advantage against lethal trypanosomiasis but also an increased risk for several chronic kidney diseases (CKD) including HIV-associated nephropathy (HIVAN). APOL1 is expressed in renal cells, however, the pathogenic events that lead to renal cell damage and kidney disease are not fully understood. The podocyte function of *APOL1-G0* versus *APOL1-G2* in the setting of a known disease stressor was assessed using transgenic mouse models. Transgene expression, survival, renal pathology and function, and podocyte density were assessed in an intercross of a mouse model of HIVAN (Tg26) with two mouse models that express either *APOL1-G0* or *APOL1-G2* in podocytes. Mice that expressed HIV genes developed heavy proteinuria and glomerulosclerosis, and had significant losses in podocyte numbers and reductions in podocyte densities. Mice that co-expressed *APOL1-G0* and HIV had preserved podocyte numbers and densities, with fewer morphologic manifestations typical of HIVAN pathology. Podocyte losses and pathology in mice co-expressing *APOL1-G2* and HIV were not significantly different from mice expressing only HIV. Podocyte hypertrophy, a known compensatory event to stress, was increased in the mice co-expressing HIV and *APOL1-G0*, but absent in the mice co-expressing HIV and *APOL1-G2*. Mortality and renal function tests were not significantly different between groups. *APOL1-G0* expressed in podocytes may have a protective function against podocyte loss or injury when exposed to an environmental stressor. This was absent with *APOL1-G2* expression, suggesting *APOL1-G2* may have lost this protective function.

## Introduction

Polymorphisms in the gene for Apolipoprotein L1 (APOL1, gene name *APOL1*) are found only in populations of recent African ancestry and confer significant risk for chronic kidney diseases (CKD) including HIV-associated nephropathy (HIVAN), idiopathic focal segmental glomerulosclerosis (FSGS), and hypertension-attributed CKD [1–5]. APOL1 is constitutively secreted into the blood and functions to kill trypanosome parasites that cause African sleeping sickness. The CKD-associated risk alleles, known as variants G1 and G2, kill a broader range of parasites compared to the common allele (known as G0) and provide an evolutionary survival advantage (reviewed in [6]). A single variant *APOL1* allele is sufficient to protect against trypanosomiasis however, risk for kidney disease is recessive requiring two variant alleles. Many individuals with a high risk genotype of two *APOL1* variants, however, do not develop kidney disease. Thus, APOL1-associated CKDs appear to be a gene-environment dependent process where the genetic susceptibility manifests in disease only when the individual is exposed to a triggering environmental stimulus.

Although the trypanolytic APOL1 in blood is abundant, studies to date have not associated circulating APOL1 with CKD risk, an observation corroborated by poorer kidney transplant outcomes dependent on donor *APOL1* genotype [7–11]. *APOL1* is also expressed in some renal cells including the podocyte [12–14]. In HIVAN and mouse models of HIVAN, HIV-1 genes also are expressed in podocytes [15–22], and HIV-1 gene expression in podocytes alone is sufficient to be disease-causing in mouse models [23, 24]. Thus, HIVAN is an ideal disease to study the functional interaction of podocyte-expressed *APOL1* with a known environmental trigger (HIV).

An intercross between APOL1 transgenic mice with a mouse model of HIVAN would provide an *in vivo* system to examine the podocyte function of *APOL1-G0* and *APOL1-G2* in the setting of a known human disease stressor. Predictions were either disease exacerbation if the *APOL1* variants contribute a deleterious function, or alternatively, disease mitigation if *APOL1-G0* provides a beneficial function. After assessment of renal function and pathology, *APOL1-G2* did not exacerbate the HIVAN phenotype. *APOL1-G0*, however, reduced podocyte losses and glomerulosclerosis suggesting *APOL1-G0* provided some protection against glomerular injury caused by HIV.

## Materials and methods

### Mouse models and phenotyping

All animal studies were conducted under oversight of Case Western Reserve University. Since APOL1 is only expressed in humans and a few other non-human primates, the use of transgenic mice expressing human *APOL1* is a tractable small animal model to study human diseases associated with *APOL1* genotype. The transgenic mouse models for podocyte-restricted expression of human *APOL1-G0* (“Tg-G0” F38 line) and *APOL1-G2* (“Tg-G2” F24 line) using the *Nphs1* promoter have matched glomerular expression patterns and were previously described [25]. The Tg26/*HIVAN4* mouse model of HIVAN is a congenic of Tg26 [26] that develops less severe kidney disease and has been previously described [27]. The *APOL1* transgenics are on the FVB/N background and the Tg26/*HIVAN4* model is >99% FVB/N with a 60Mb BALB/c-derived genomic region referred to as the *HIVAN4* locus [27]. The Tg26/*HIVAN4* and *APOL1* transgenic models are maintained as carriers (hemizygotes), thus the intercross generated all possible single and dual transgenics for age-matched comparisons. The *APOL1* transgenic mice cannot be bred to carry two copies of the transgene due to an unrelated phenotype on pregnancy [25] that becomes pragmatically difficult to maintain the lines as homozygotes. Mice were housed in a specific pathogen free conventional animal facility and standard breeding practices were used to generate F1 hybrids resulting in the expected Mendelian proportions of: 25% non-transgenic (wildtype), 25% *APOL1* single transgenic, 25% Tg26/*HIVAN4* single transgenic, and 25% *APOL1* plus Tg26/*HIVAN4* dual transgenic mice. All dual transgenics carried a single copy of the respective *APOL1* gene similar to the single transgenics.

Two hundred day-old F1 hybrids (n = 18–21 each group, combined males and females) were phenotyped for kidney disease typical of HIVAN. Renal function testing was performed by the Vanderbilt Center for Kidney Disease Pathology and Phenotyping Core and included ELISAs for urinary albumin and creatinine and HPLC assays for serum creatinine. Kidneys were PAS stained and glomerular and tubular pathology was scored using quantitative methods by pathologists blinded to sample identity. Total number of glomeruli were counted, and percentages of glomeruli with the following features were calculated: segmental sclerosis, global sclerosis, segmental collapse, global collapse, podocyte hypertrophy (of ≥1 podocyte with large/prominent cell body with or without increased size of the nucleus), glomerular hyperplasia (potentially of parietal cell or podocyte origin, of ≥2 layers of normal or hypertrophic podocytes). Tubular microcysts (dilated tubule, often with serpiginous appearance, containing a large hyaline cast), tubular atrophy, interstitial fibrosis, and interstitial inflammation also were scored using a semi-quantitative scale of 0 to 4, where 0 = unaffected; 0.5 = 1–5% affected; 1 = 6–25% affected; 2 = 26–50% affected; 3 = 51–75% affected; 4 = >75% affected.

### Imaging and podocyte density

Kidney sections were examined using immunofluorescence and confocal microscopy as described previously [14]. Primary antibodies used were mouse monoclonal Synaptopodin (1:10 dilution, BioDesign) and rabbit polyclonal WT-1 (1:200 dilution, Santa Cruz Biotech). Podocyte counts, glomerular volumes, and podocyte density calculations used a method originally described by Venkatareddy *et al*. [28] and as used previously for characterization of the Tg-G0 and Tg-G2 mouse models [25].

### Quantitative PCR

Kidneys from APOL1, *HIVAN4* single and dual transgenic mice were used for glomeruli isolation followed by RNA extraction (Qiagen microRNA kit), reverse transcription (Roche AMV cDNA synthesis kit), and quantification using 1μg of cDNA in real time PCR (Applied Biosystems Quantstudio 5 PCR System and Power SYBR Green PCR Master Mix) using the ΔCt method with Tubulin as the reference. Primers used were: *Tuba1a* (internal control, forward: TGCCTTTGTGCACTGGTATG, reverse: CTGGAGCAGTTGACGACAC), *APOL1* (forward: TCGTGGCTGCTGCTGAACTG, reverse: GCGATGGTGGTGCCTTTGTG), *Nphs1* (forward: AGCTACCCTGCATAGCCAGA, reverse: ACCCTCCAGTTAACTTGCTTTGG*)*, HIV *nef* (forward: GGTGGGTTTTCCAGTCACAC, reverse: GGGAGTGAATTAGCCCTTCC).

### Statistical methods

Podocyte density calculations were based on ~50 glomeruli for each animal with combined male and female mice per group (actual group numbers are in figure legends). Group differences were analyzed using ANOVA. Group differences for renal function tests were analyzed using Kruskal-Wallace. Generalized linear mixed models and Markov Chain Monte Carlo samples were used to evaluate differences in podocyte density, glomerular volume, and corrected podocyte count between groups. Differences in renal function tests and histopathology scoring between groups were determined by *t* test followed by Bonferroni correction. *P* values ≤0.05 were considered significant. Animals that died or were euthanized for humane reasons prior to the 200 day endpoint were included in the analysis. Inclusion of these animals did not alter study outcomes by sensitivity analyses. Primary data for all statistical comparisons are provided in S1 File.

## Results

Two transgenic mouse models expressing either the human *APOL1-G0* (“Tg-G0”) or *APOL1-G2* (“Tg-G2”) genes under control of the Nephrin (*Nphs1*) promoter, restrict *APOL1* expression to podocytes. The G0 and G2 transgenic mice do not spontaneously develop CKD, but develop an unrelated phenotype resembling preeclampsia that reduces fecundity [25]. Since mice do not have an ortholog of human *APOL1*, the Tg26/*HIVAN4* mice would represent an *APOL1* null phenotype, and mice expressing *APOL1-G0* would functionally recreate a human CKD low risk *APOL1* genotype, whereas mice expressing *APOL1-G2* would functionally recreate a human CKD high risk *APOL1* genotype. Although a human G2 high risk genotype is the carriage of two G2 alleles, our *APOL1* transgenic mice carry only a single G2 allele. However, like humans with a G2 high risk genotype, the transgenic mice only express *APOL1* G2 in their podocytes. The Tg26/*HIVAN4* mouse model of HIVAN is transgenic for a subgenomic HIV-1 provirus and spontaneously develops a progressive and lethal kidney disease that replicates most of the pathology and clinical presentation of the human disease [26, 27]. F1 hybrids from intercrossing Tg26/*HIVAN4* with Tg-G0 or Tg-G2 (dual transgenics referred to as “Tg26+G0” and “Tg26+G2”) were examined at 200 days of age for pathology and renal function using parameters previously established to quantitate disease severity in the Tg26 model [29]. Age-matched non-transgenic (wildtype), Tg26/*HIVAN4*, and Tg-G0 and Tg-G2 single transgenics were also examined as comparators.

### Renal pathology

**Fig 1:**
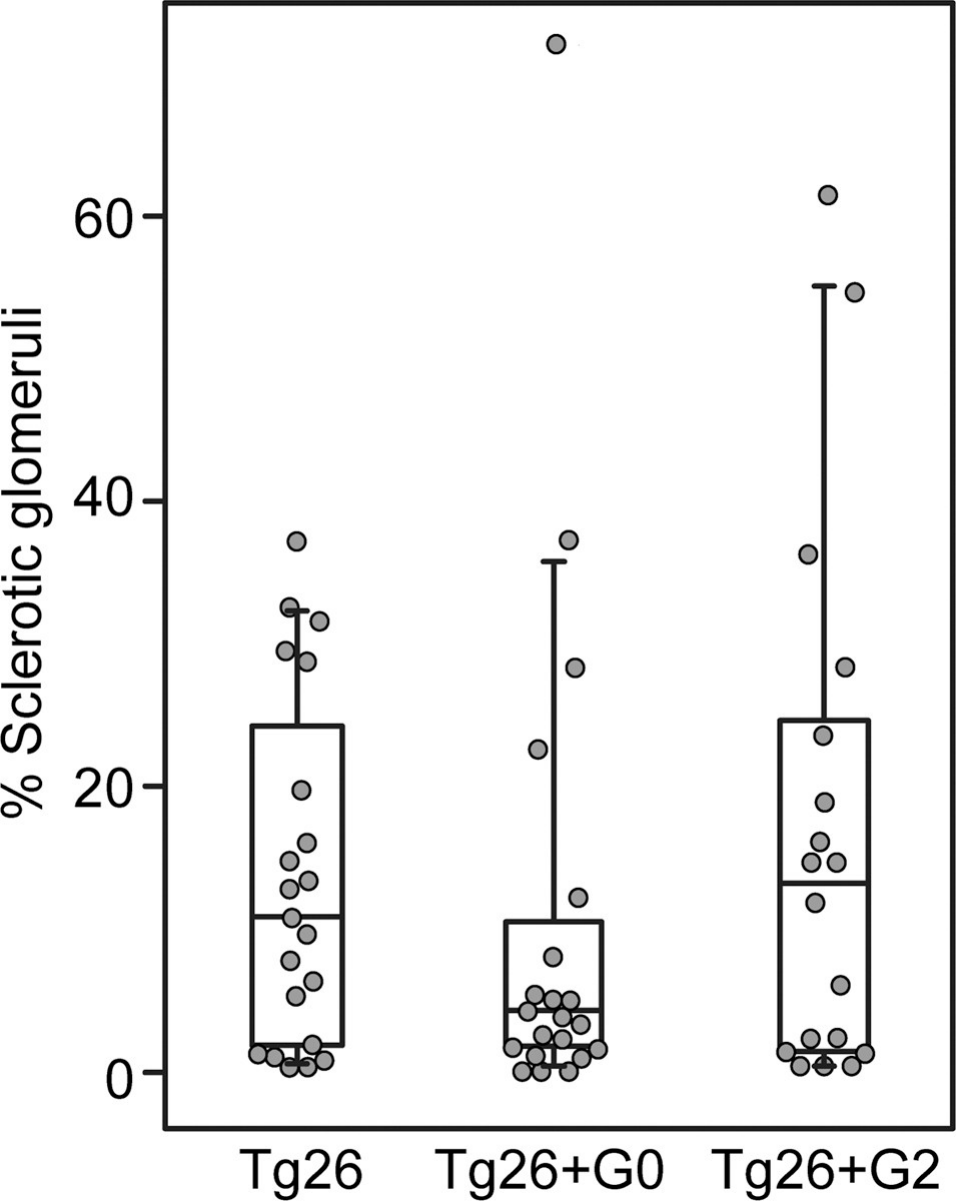
*APOL1-G0* reduced glomerulosclerosis in a murine model of HIVAN. Box whisker plot of total sclerotic glomeruli (aggregate global and sclerotic glomeruli from Table 1) as a percentage of total scored glomeruli per animal. Each data point represents one mouse, boxes are interquartile range, whiskers are 95% confidence intervals. Numbers in each group were Tg26 n = 21, Tg26+G0 n = 21, Tg26+G2 n = 18.

Two pathologists, blinded to sample identity, independently assessed (scored) ten different parameters of glomerular and tubulointerstitial damage (**Table 1**). All tubulointerstitial features were not statistically different between groups. Several individual glomerular features, however, were different. The percentage of sclerotic glomeruli trended lower in the Tg26+G0 hybrid mice (**Fig 1**). In addition, podocyte hypertrophy was greater in Tg26+G0 mice compared to Tg26+G2 mice (**Table 1**). Only one Tg26+G2 mouse exhibited glomeruli with hypertrophic podocytes, whereas hypertrophy was evident in glomeruli of over half of the Tg26+G0 mice.

**Table 1:** Histopathology of Tg26 and Tg26 x APOL1 dual transgenic mice.

	Tg26 (n = 21)	*P*	Tg26+G0 (n = 21)	*P*	Tg26+G2 (n = 18)	*P*
# scored glomeruli per animal, mean	201		230		210	
Segmental sclerotic, % of total glomeruli	9.9%	ref	6.6%	NS	10.9%	NS
Global sclerotic, % of total glomeruli	3.7%	ref	3.8%	NS	5.2%	NS
Segmental collapse, % of total glomeruli	0%	ref	0%	NS	0%	NS
Global collapse, % of total glomeruli	0%	ref	0.4%	NS	0%	NS
Glomerular hyperplasia, any	6.0%	ref	6.0%	NS	1.5%	NS
Podocyte hypertrophy, any	10.0%	ref	16.0% *	NS	1.5%	NS
Tubular microcysts, mean±SD scored on 0–4 scale	0.88 ± 1.27	ref	0.90 ± 1.47	NS	0.89 ± 1.33	NS
Tubular atrophy, mean±SD scored on 0–4 scale	0.07 ± 0.24	ref	0.05 ± 0.22	NS	0 ± 0	NS
Interstitial fibrosis, mean±SD scored on 0–4 scale	0.05 ± 0.22	ref	0.20 ± 0.52	NS	0.17 ± 0.51	NS
Interstitial inflammation, mean±SD scored on 0–4 scale	0.93 ± 1.29	ref	1.26 ± 1.66	NS	1.08 ± 1.41	NS

NS, not significant compared to Tg26. * *P* = 0.03 compared to Tg26+G2.

### Podocyte density

The podocyte depletion hypothesis purports chronic glomerular diseases are mediated by progressive podocyte losses via podocyte detachment or death. It has been validated in many human and rodent disease models including the age-related decline in renal function (reviewed in [30]). Although the Tg-G0 and Tg-G2 transgenic mice do not spontaneously develop kidney disease, the Tg-G2 mice have an accelerated age-related decrease in podocyte densities compared to Tg-G0 or wild-type mice [25]. This accelerated podocyte loss is not associated with podocyte cell death and remained subclinical, with longitudinal predictions that podocyte attrition would remain insufficient to initiate glomerulosclerosis through the average mouse lifespan [31]. It is unknown whether this accelerated podocyte depletion could be exacerbated by a disease stressor, and thus, underlie a pathogenic mechanism of the *APOL1* variants.

**Fig 2:**
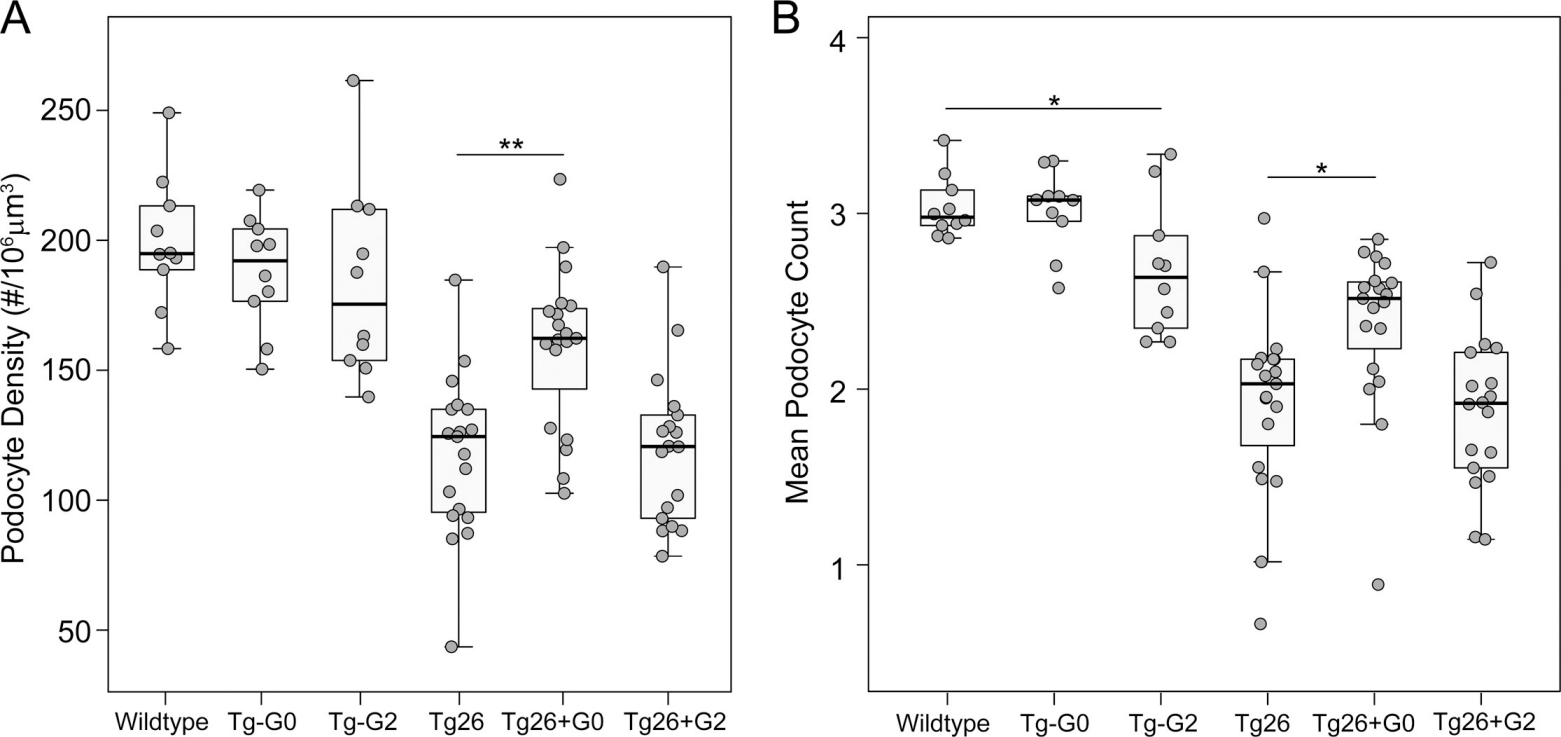
*APOL1-G0* preserves podocyte density in a murine model of HIVAN. **A**. Mean podocyte densities calculated from podocyte counts (panel B) and glomerular volumes (S2 Fig). **B**. Mean podocyte number per glomeruli. Each data point represents one mouse, boxes are interquartile range, whiskers are 95% confidence intervals. Numbers in each group were: Non-transgenic (wild-type) n = 10, Tg26 n = 19, Tg26+G0 n = 19, Tg26+G2 n = 18, Tg-G0 n = 10, Tg-G2 n = 10. Statistical comparisons were made to the relevant non-*APOL1* expressing group (Tg-G0 or Tg-G2 versus wildtype, and Tg26+G0 or Tg26+G2 versus Tg26; *P<0.05, **P<0.01).

In Tg26/*HIVAN4* mice, podocyte densities were significantly less compared to all other non-diseased groups as would be expected for the progressive glomerular disease that occurs in the model at 200 days of age. Podocytes were lost segmentally, exhibiting losses in WT-1 positivity (podocyte number) but with preserved Synaptopodin staining (glomerular volume), a finding that reflects possible compensatory hypertrophy by the residual podocytes. More severely affected glomeruli exhibited losses in both WT-1 positivity and Synaptopodin staining, resulting in reductions in both podocyte number and glomerular volume which occurs when podocyte loss exceeds the adaptive capacity of the remaining podocytes (**S1 Fig**). Podocyte densities in the dual transgenic Tg26+G0 and Tg26+G2 were both significantly reduced compared to the single transgenic Tg-G0 and Tg-G2 mice due to the glomerulosclerosis of the Tg26/*HIVAN4* model (**Fig 2A**). Podocyte densities in the Tg26/*HIVAN4* and Tg26+G2 dual transgenic mice were not significantly different. However, podocyte densities in the Tg26+G0 dual transgenics were significantly greater than either the Tg26+G2 dual transgenic and Tg26/*HIVAN4* mice. This preservation of podocyte density was driven by higher numbers of podocytes (**Fig 2B**) since glomerular volumes were not significantly different (**S2 Fig**). This suggests podocyte *APOL1-G0* expression functioned to reduce podocyte loss in the setting of HIVAN-like kidney disease.

### Renal function

Standard renal function tests for serum creatinine and urinary albumin to creatinine ratios were not significantly different in any of the groups (**Table 2**). As expected, some animals died from renal failure or reached predetermined humane endpoints and were sacrificed before study end, but there was no significant difference in survival in any group (**S3 Fig**).

**Table 2:** Renal function in single and dual transgenic mice.

	n (%male)	Serum Creatinine/ body weight (mg/dl/kg) mean ± SD	*P*	UACR (μg/mg) median (IQR)	*P*
**Tg26**	21 (71%)	4.2 ± 0.9	ref	212 (84,493)	ref
**Tg26+G0**	21 (62%)	6.8 ± 5.2	NS	141 (108, 226)	NS
**Tg26+G2**	18 (33%)	4.2 ± 1.3	NS	119 (97, 179)	NS

UACR, urinary albumin to creatinine ratio. SD, standard deviation. IQR, interquartile range.

NS, not significant.

### Transgene expression

**Fig 3:**
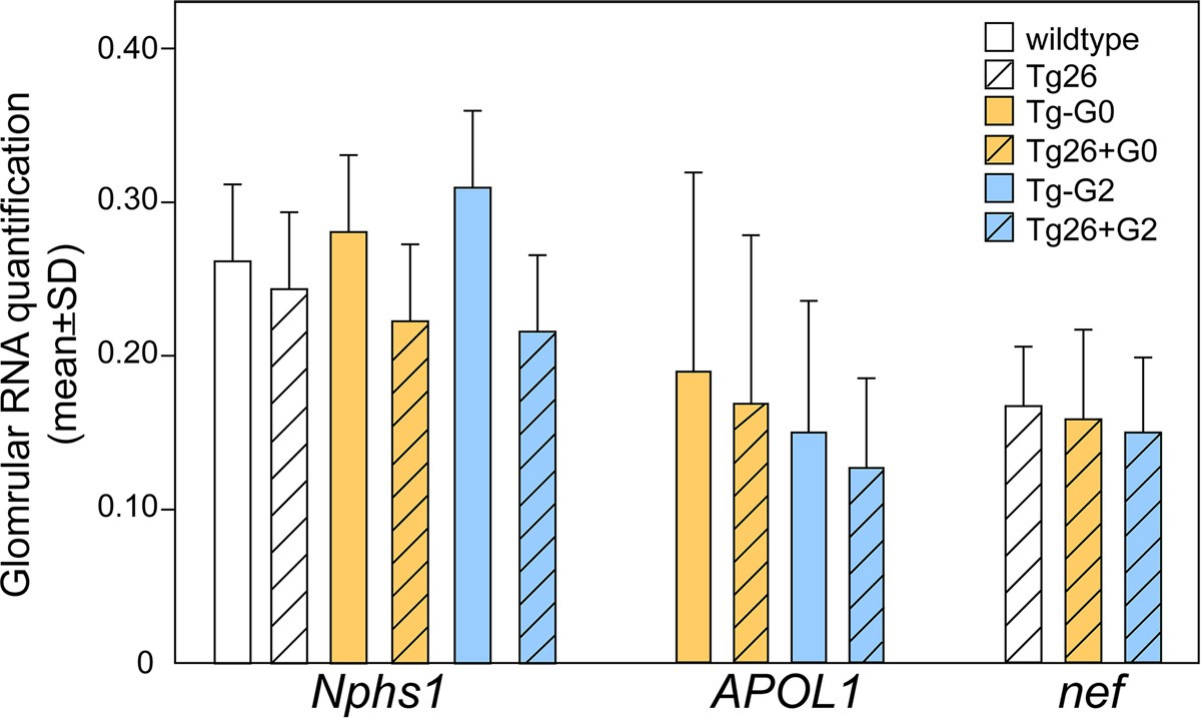
Transgene expression levels were not altered in dual transgenic mice. RNA from isolated glomeruli were quantified from normal (wildtype) n = 6, Tg26 n = 8, Tg-G0 n = 7, Tg-G2 n = 5, Tg26+G0 n = 6, Tg26+G2 n = 7. *Nphs1* (Nephrin) expression was used as a reference podocyte gene. Data are 1/ΔCt normalized to *Tuba1a* (Tubulin) levels.

Since the Tg26 HIVAN model phenotype is dependent on HIV transgene expression, we evaluated both APOL1 and HIV transgene expression to verify levels were not significantly different in the dual transgenic mice (Fig 3). Quantification of RNA extracted from isolated glomerular from single and dual transgenic mice was not significantly different between groups. There was a trend toward lower levels of *Nphs1* with expression of the HIV transgene, possibly reflecting the loss of podocyte differentiation that has been previously described for this mouse model and human HIVAN [32]. The expression levels of the HIV transgene was quantified using the *nef* transcript, as expression of Nef is strongly linked with HIVAN pathology in this model [33], and was not different between groups. Expression of *APOL1* was not significantly different between groups. These studies would indicate the expression of one transgene did not alter the expression of the other transgene in dual transgenics, and the observed differences in renal phenotypes were likely not related to significant differences in transgene expression.

## Discussion

The mechanism by which the *APOL1* variants cause CKD remains unclear, and an unresolved issue is whether the *APOL1* variants are gain-of-function or loss-of-function mutations. There were no significant differences between the CKD phenotypes of the Tg26/*HIVAN4* model with co-expression of G2, indicating our prior report of an accelerated age-associated loss of podocytes in an unstressed state for the Tg-G2 mouse model was not reflecting a disease process that could be exacerbated with a stressor. On the contrary, the observed G0-dependent preservation of podocyte numbers and reduced glomerulosclerosis in the Tg26+G0 phenotype suggests G0 may be providing a mechanism to reduce stress-induced podocyte losses.

This preservation of podocytes may be related to podocyte hypertrophy, the only other significant difference between Tg26+G0 and Tg26+G2 mouse glomeruli. In glomerular disease, podocyte hypertrophy is a compensatory mechanism that maintains glomerular tuft coverage and preserves filtration barrier function in response to podocyte injury and loss [34, 35]. After podocyte loss, the remaining healthy podocytes hypertrophy to cover the vacant capillary surface. The observation that G0 mice had enhanced podocyte hypertrophy may suggest G0 function is involved in this compensatory stress response. The absence of hypertrophied podocytes in the Tg26+G2 mice may reflect either an absence of a hypertrophic response, or alternatively, the Tg26+G2 podocytes may have hypertrophied but then detached from the tuft and were lost. Studies of APOL1 function in *Drosophila* observed nephrocytes expressing G0 or G1 progressively hypertrophied and died as the fly aged, and this response was greater with G1 expression [36]. If hypertrophy and cell loss is exaggerated with risk variant expression, additional hemodynamic factors [37] or differences in cell-cell or cell-matrix attachment [38] that occur in disease may contribute to the enhanced podocyte depletion.

Since APOL1 is only present in humans and a few other primates, transgenic mouse models are a pragmatic method to assess whole animal physiology of APOL1 function. However with any model system, there are limitations. The most significant concern is whether the human cellular pathways involving APOL1 function are present in mice. In addition, our mouse models restrict *APOL1* expression using the Nephrin promoter to podocytes and does not replicate the induction of *APOL1* expression by immune mediators and does not replicate expression in other sites, most notably expression in renal endothelium and in circulation. In our study, there was no significant effect on proteinuria, despite preservation of podocytes with reduced glomerulosclerosis. This may suggest additional pathogenic events in kidney cells other than podocytes may be important overall contributors to APOL1-associated CKD, which are not recreated in our mouse models of podocyte-restricted *APOL1* expression. Newly developed transgenic mouse models that express the entire *APOL1* gene including the flanking regulatory regions would be a better system to fully evaluate the stress-associated functions of APOL1 in CKD.

This study is the first *in vivo* test of the function of kidney-expressed *APOL1* concurrent with a known human disease stressor. APOL1 expressed in the kidney may provide resilience to podocytes to tolerate disease stresses. This stress-related function of *APOL1* was only evident with G0, and not G2, indicating the *APOL1* risk variants may have lost this function related to podocyte preservation. These studies would suggest a logical approach for APOL1 targeted therapies would be to restore APOL1-G0 function in subjects with a high risk genotype.

## Supporting information

S1 Fig Podocyte depletion occurs in the Tg26/*HIVAN4* mouse model. (PDF)

S2 Fig Podocyte density losses were not dependent on glomerular volume changes. (PDF)

S3 Fig No differences in survival rates of dual transgenics compared to Tg26/*HIVAN4*. (PDF)

S1 File Primary data sets for clinical and pathological scoring, podocyte density calculations, and quantitative PCR. (XLSX)
