# Supplementary material for: APOL1-G0 protects podocytes in a mouse model of HIV-associated nephropathy
Source: PLoS One. 2019 Oct 29;14(10):e0224408. doi: 10.1371/journal.pone.0224408 (PMC6818796; doi:10.1371/journal.pone.0224408)
Supplement: S1 Fig — (PDF) [file pone.0224408.s001.pdf]

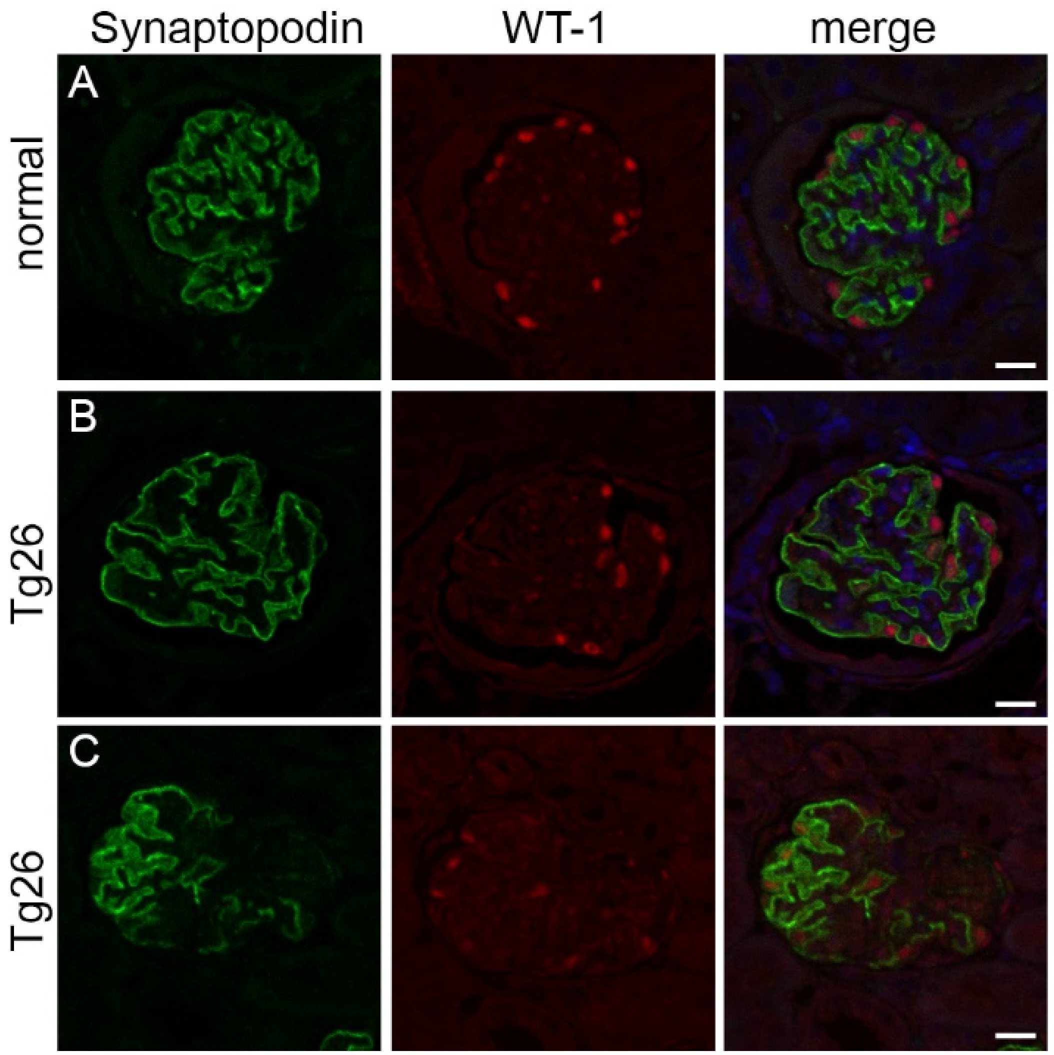

Supplemental Figure 1. Podocyte depletion occurs in the Tg26/*HIVAN4* mouse model. Immunofluorescence staining of WT-1 (nuclear) and Synaptopodin (cytoplasmic) in 200 day-old mouse glomeruli used to enumerate podocytes and estimate glomerular volumes respectively. **A**. Glomerulus from a normal mouse with typical distribution of podocytes and glomerular tuft structure. **B**. Glomerulus from a Tg26/*HIVAN4* mouse with a segmental loss of WT-1 positive nuclei, indicating loss of podocytes, but with uniform Synaptopodin positivity, indicating preservation of coverage of the tuft capillary surface (i.e. hypertrophy). **C**. Glomerulus from a Tg26/*HIVAN4* mouse with more advanced glomerular disease exhibiting both losses in number of WT-1 positive nuclei (and a qualitative reduced intensity of WT-1 staining), and segmental losses in Synaptopodin staining. Merge panel includes DAPI nuclear stain (blue). Scale bar=25µm.
