# Supplementary material for: APOL1-G0 protects podocytes in a mouse model of HIV-associated nephropathy
Source: PLoS One. 2019 Oct 29;14(10):e0224408. doi: 10.1371/journal.pone.0224408 (PMC6818796; doi:10.1371/journal.pone.0224408)
Supplement: S2 Fig — (PDF) [file pone.0224408.s002.pdf]

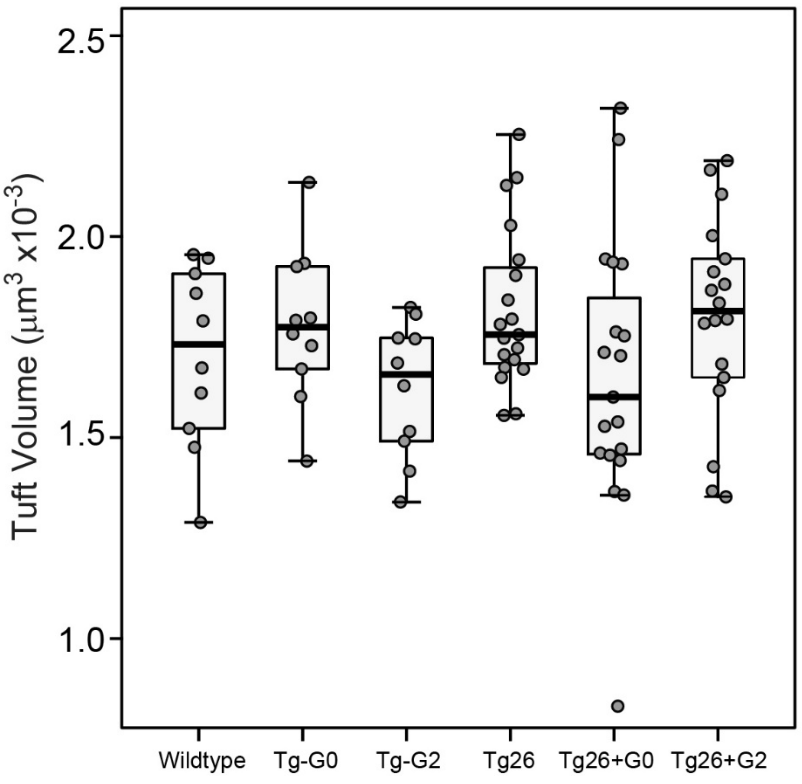

Supplemental Figure 2. Podocyte density losses were not dependent on glomerular volume changes. Glomerular tuft volumes were not significant differences between groups. Statistical comparisons were made to the relevant non-*APOL1* expressing group (Tg-G0 or Tg-G2 versus wildtype, and Tg26+G0 or Tg26+G2 versus Tg26). Each data point represents one mouse.
