# Supplementary material for: APOL1-G0 protects podocytes in a mouse model of HIV-associated nephropathy
Source: PLoS One. 2019 Oct 29;14(10):e0224408. doi: 10.1371/journal.pone.0224408 (PMC6818796; doi:10.1371/journal.pone.0224408)
Supplement: S3 Fig — (PDF) [file pone.0224408.s003.pdf]

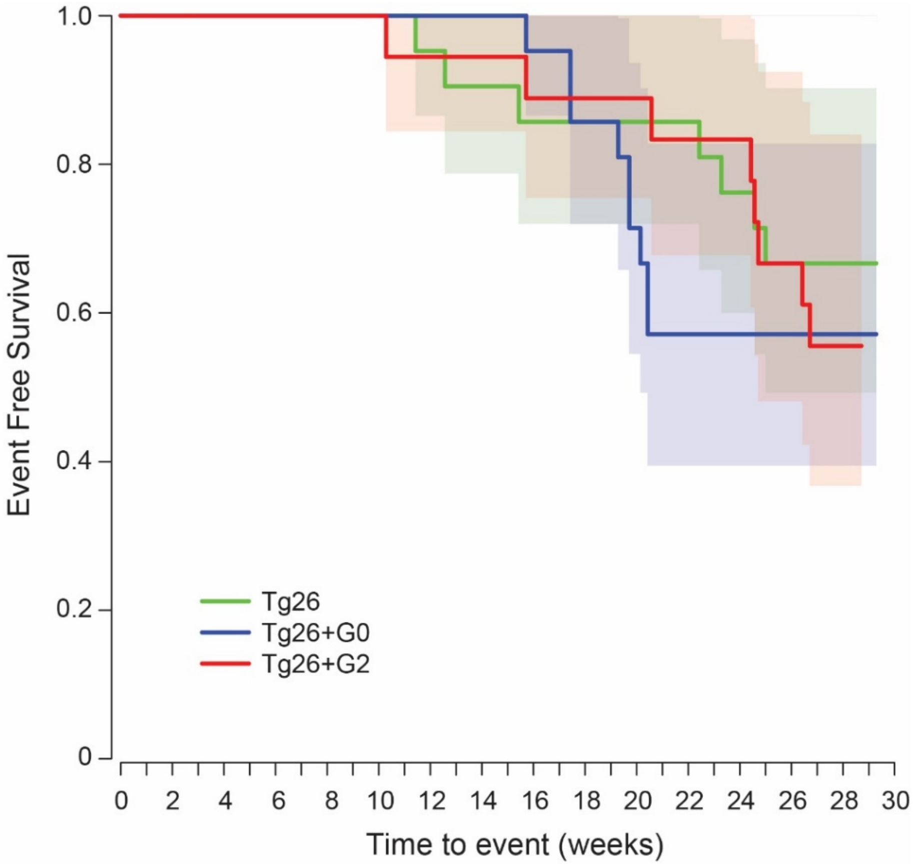

Supplemental Figure 3. No differences in survival rates of dual transgenics compared to Tg26/*HIVAN4*. Kaplan Meier plot of animals that died or reached one of the predetermined humane endpoints (this included renal failure or skin lesions that are typical of the Tg26 mouse model) prior to study endpoint of 200 days of age (29 weeks). Data are number of deaths (solid line) ± standard deviation (shaded area). There was no significant difference between groups.
